# Molecular Regulation of Biofilm Development in *Stenotrophomonas maltophilia*: Integrating Signal Transduction, Environmental Adaptation, and Antibiotic Resistance

**DOI:** 10.3390/pathogens15080874

**Published:** 2026-08-20

**Authors:** Ke Yu, Gexiao Zhao, Qing Zhang, Xiaobing Zhang

**Affiliations:** 1 Department of Laboratory Medicine, The First Affiliated Hospital of Chongqing Medical University, Chongqing 400016, China; 17828295690@163.com (K.Y.); 18982750647@163.com (Q.Z.); 2 Department of Laboratory Medicine, Sichuan University West China Hospital Guang’an Hospital, Guang’an People’s Hospital, Guang’an 638001, China; peaopao@foxmail.com

**Keywords:** *Stenotrophomonas maltophilia*, biofilm formation, multidrug resistance, quorum sensing, efflux pumps

## Abstract

*Stenotrophomonas maltophilia* is increasingly recognized as a difficult-to-treat healthcare-associated opportunistic pathogen, particularly in critically ill and immunocompromised patients, in whom it causes severe respiratory, bloodstream, and device-associated infections. Its intrinsic resistance to multiple antimicrobial classes, capacity to acquire additional resistance determinants, and ability to establish persistent biofilms substantially limit therapeutic options. This review integrates current knowledge of the structural basis, regulatory circuitry, and ecological interactions governing *S. maltophilia* biofilm development and examines how these processes converge with antimicrobial resistance. Biofilm formation is driven by coordinated adhesion and motility, extracellular matrix production, quorum sensing, cyclic di-GMP signaling, two-component regulatory systems, and adaptive responses to iron limitation and oxidative stress. Multidrug efflux systems contribute not only to antibiotic extrusion but also to membrane homeostasis, motility, stress adaptation, and biofilm-associated phenotypes, thereby providing a functional link between antimicrobial resistance and bacterial persistence. In polymicrobial communities, interspecies signaling and competitive or cooperative interactions further reshape biofilm architecture and antimicrobial tolerance. Collectively, current evidence indicates that *S. maltophilia* biofilm formation arises from interconnected regulatory networks rather than isolated molecular determinants. Targeting matrix assembly, signaling pathways, stress adaptation, or resistance-associated physiology may therefore complement conventional antimicrobial therapy. Future studies should prioritize clinically representative isolates, physiologically relevant multispecies models, and in vivo validation to translate mechanistic insights into effective anti-biofilm interventions.

## 1. Introduction

*Stenotrophomonas maltophilia* (*S. maltophilia*) is an obligately aerobic, Gram-negative rod that possesses polar flagella, enabling motility and facilitating environmental adaptation. It is widely distributed in diverse ecological niches, including soil, plant rhizospheres, aquatic environments, hospital settings, and wastewater treatment systems [1]. Once considered an environmental microorganism with limited clinical significance, *S. maltophilia* has emerged as an important opportunistic pathogen. Although its intrinsic virulence is relatively low, it readily adheres to respiratory epithelial cells and indwelling medical devices, promoting persistent colonization and biofilm formation that can lead to severe infections. Over recent decades, the clinical recognition and healthcare burden of *S. maltophilia* infections have increased, particularly among patients requiring intensive care, prolonged mechanical ventilation, broad-spectrum antimicrobial therapy, or invasive medical support. Over recent decades, the incidence of healthcare-associated infections caused by *S. maltophilia* has increased substantially. It is now recognized as a major cause of bloodstream infections, pneumonia, and other device-associated infections, particularly among immunocompromised and critically ill patients. Recent multicenter evidence further highlights the severity of invasive disease: among 321 patients with *S. maltophilia* bloodstream infection treated at ten hospitals, the 30-day all-cause mortality rate was 46.1% [2]. Reported predictors of mortality include intensive care unit (ICU) admission, central venous catheterization, mechanical ventilation, underlying malignancy, delayed catheter removal, inappropriate antimicrobial therapy, thrombocytopenia, and septic shock [2,3].

Recent epidemiological evidence suggests that the clinical burden and resistance patterns of *S. maltophilia* are heterogeneous across patient populations, healthcare settings, and geographical regions, rather than increasing uniformly worldwide. A national surveillance study analyzing 514,768 clinical isolates collected from 1412 medical institutions in China between 2014 and 2021 found that *S. maltophilia* accounted for approximately 2.1% of bacterial isolates overall, with disproportionately higher prevalence among ICU and older patients. Importantly, the observed temporal trends were specific to the Chinese surveillance setting: resistance to trimethoprim–sulfamethoxazole decreased from 9.8% to 7.5%, whereas levofloxacin resistance increased modestly from 8.5% to 9.5% [4]. These trends should not be extrapolated directly to other geographical regions. At the global level, a systematic review and meta-analysis of 289 studies confirmed extensive intrinsic resistance to several antimicrobial classes, particularly carbapenems, while demonstrating substantial geographical variation in susceptibility to clinically relevant agents [5]. The inclusion of a dedicated section on *S. maltophilia* in the 2024 Infectious Diseases Society of America guidance on antimicrobial-resistant Gram-negative infections further reflects the growing clinical importance of this difficult-to-treat pathogen and the limited evidence available to define a standard therapeutic regimen [6].

Biofilms are highly organized microbial communities embedded within an extracellular polymeric substance (EPS) matrix composed of microbial products together with materials derived from the surrounding environment or host [7]. This matrix, composed primarily of extracellular polysaccharides, proteins, extracellular DNA (eDNA), and lipids, is essential for maintaining the three-dimensional biofilm architecture, facilitating intercellular communication, and enhancing bacterial adaptation to environmental stresses [8]. In *S. maltophilia*, biofilm formation is closely associated with antimicrobial resistance and persistent infection. Biofilm formation is particularly relevant to persistent respiratory colonization and infections involving indwelling medical devices, including central venous catheters, endotracheal tubes, and prosthetic materials. Rather than acting solely as a passive physical barrier, the biofilm environment can reduce antimicrobial effectiveness through restricted penetration of some agents, altered bacterial metabolism, enrichment of persister-like subpopulations, activation of stress-response pathways, and protection from host immune clearance. These processes primarily confer biofilm-associated antimicrobial tolerance, whereas prolonged bacterial survival under antimicrobial pressure may facilitate the selection and maintenance of genetically resistant populations [9].

**Figure 1:**
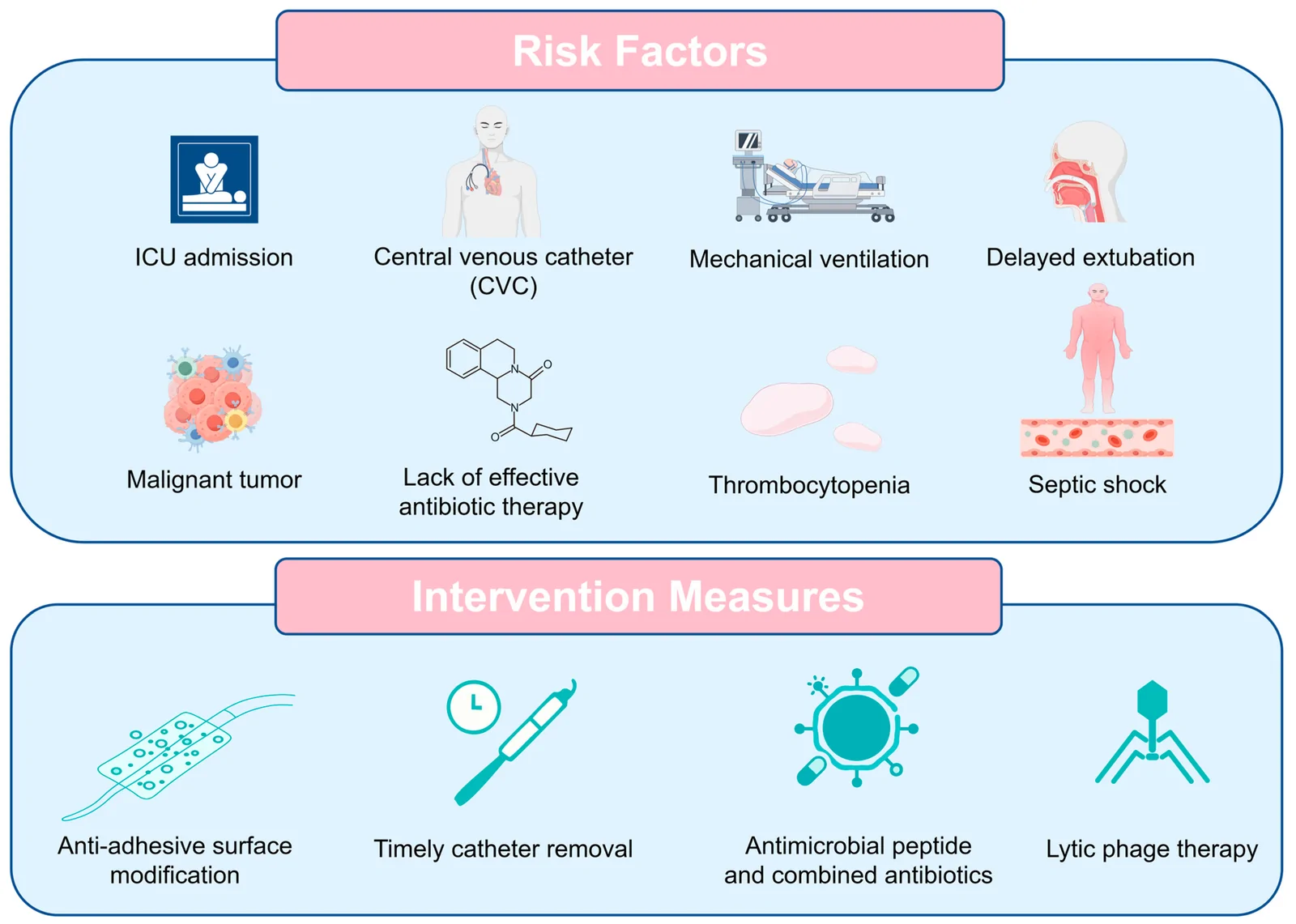
Clinical burden, poor-outcome-associated factors, and potential biofilm-oriented intervention strategies in *S. maltophilia* infection. By Figdraw.

Consequently, biofilm formation and antimicrobial resistance should be regarded as interconnected but mechanistically distinct contributors to persistent colonization, recurrent infection, and therapeutic failure (Figure 1).

Throughout this review, we distinguish mechanisms directly demonstrated in *S. maltophilia* from those inferred from homologous systems or broader bacterial biofilm studies. Where direct species-specific evidence remains limited, this distinction is explicitly stated.

**Figure 2:**
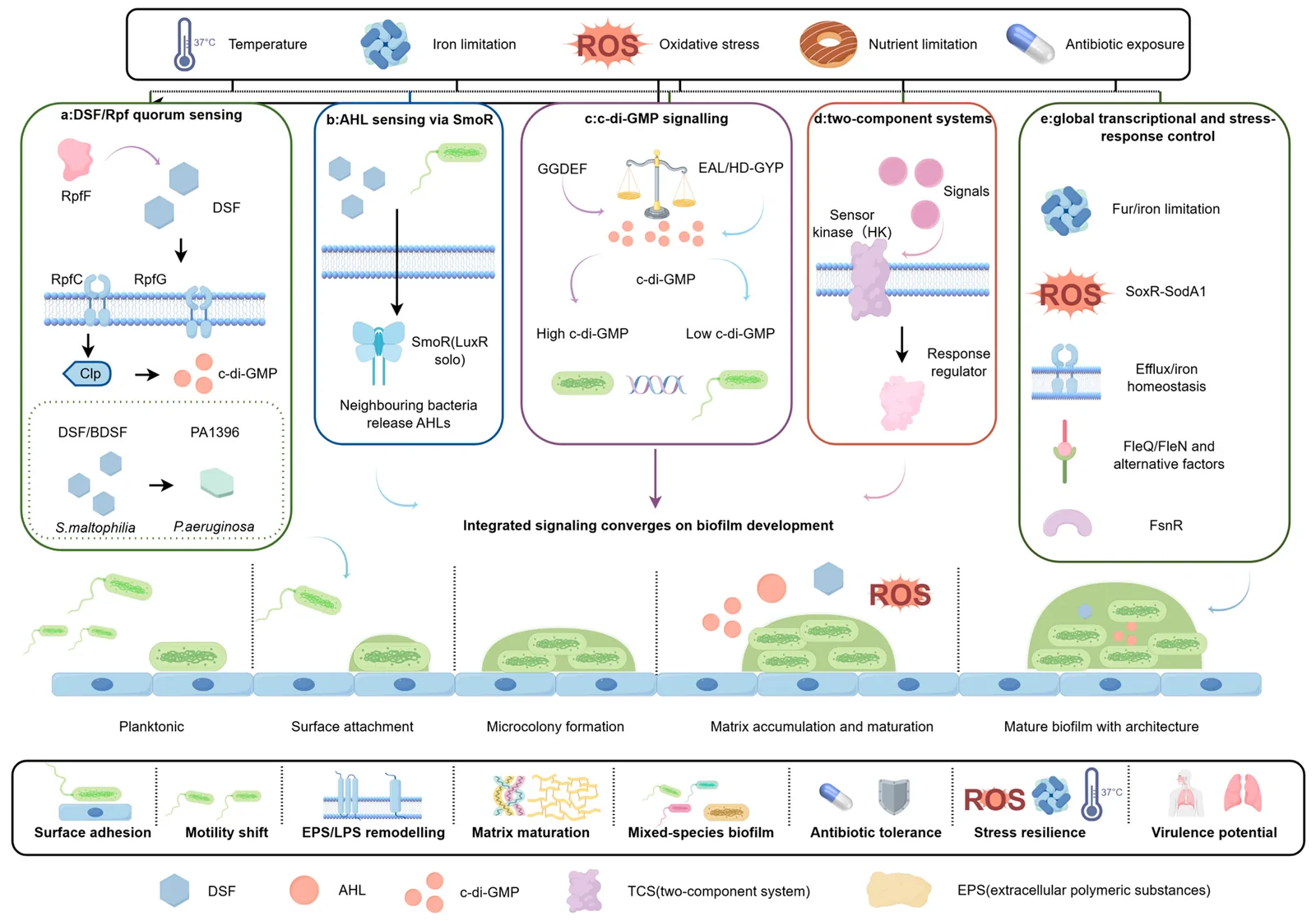
Integrated regulatory framework governing biofilm development in *Stenotrophomonas maltophilia*. (**a**) The DSF (diffusible signal factor)/rpf quorum-sensing system regulates biofilm formation through RpfF-dependent DSF production and RpfC/RpfG-mediated signal transduction, with additional interspecies effects on Pseudomonas aeruginosa. (**b**) The LuxR-solo SmoR senses exogenous AHL (N-acyl homoserine lactones) signals released by neighboring bacteria and modulates behaviors related to swarming, colonization, and biofilm development. (**c**) c-di-GMP (cyclic diguanylate monophosphate) signaling acts as a central molecular switch in biofilm regulation, where elevated c-di-GMP promotes adhesion, EPS production, and biofilm maturation, whereas reduced c-di-GMP favors motility and dispersal. (**d**) Two-component systems transmit environmental cues through sensor kinases and response regulators, thereby linking external stimuli to transcriptional programs involved in biofilm development. (**e**) Global transcriptional and stress-response networks, including iron limitation, oxidative stress, nutrient limitation, temperature change, and antibiotic exposure, further shape biofilm formation through regulators such as Fur, SoxR-SodA1, efflux-associated homeostasis pathways, FleQ/FleN, alternative sigma factors, and FsnR. By Figdraw.

Given the growing clinical challenge posed by antimicrobial-resistant *S. maltophilia*, this review systematically examines the structural determinants and regulatory networks governing biofilm initiation, maturation, and persistence and evaluates how these processes intersect with antimicrobial resistance and interspecies interactions. By integrating current molecular evidence with its ecological, epidemiological, and clinical context, this review aims to provide a comprehensive framework for understanding biofilm-associated persistence and to identify potential therapeutic targets for the development of clinically relevant anti-biofilm strategies (Figure 2).

## 2. Stage-Specific Processes and Structural Basis of *S. maltophilia* Biofilm Formation

### 2.1. Initial Adhesion: Roles of Flagella, Pili, and Adhesins

For *S. maltophilia*, flagella can assist early surface attachment, yet they barely serve as the decisive factor for mature biofilm total biomass. One study showed that clinical isolates from patients with cystic fibrosis adhered to IB3-1 epithelial monolayers and formed typical biofilm structures, as confirmed by scanning electron microscopy and confocal microscopy. Loss of flagella significantly reduced bacterial adhesion (*p* < 0.001), whereas the internalization rate into IB3-1 cells remained low, ranging from 0.01% to 4.94% [10]. Previous research by de Oliveira-Garcia’s team verified that this bacterium synthesizes flagella when expanding on inert materials, and the purified flagellum consists of a 38 kDa protein subunit named SMFliC [1]. However, assays comparing swimming and twitching motility among non-respiratory strains and cystic fibrosis isolates found no clear correlation between motility and biofilm formation [11]. Correspondingly, transmission electron microscopy with negative staining and agar mobility tests proved that fliF gene knockout strains without flagella could form PVC biofilms whose adhesion ability and total mass were similar to wild strains. Collectively, these findings suggest that flagella contribute particularly to initial surface approach, adhesion, and early biofilm organization, whereas their contribution to mature biofilm biomass appears to be context dependent. The apparently discordant findings among studies may reflect differences in strain background, clinical origin, growth substrate, nutrient conditions, incubation time, and the experimental endpoints used to quantify biofilm formation. Therefore, flagellar function should not be considered uniformly dispensable for mature biofilm development; rather, its relative contribution appears to vary across experimental and biological contexts.

Surface appendages contribute to different but partially overlapping stages of *S. maltophilia* biofilm development. *SMF-1* fimbriae have the strongest evidence for a role in initial attachment: they mediate adherence to epithelial cells and abiotic surfaces, and antibodies against *SMF-1* reduce biofilm formation [12]. Consistently, disruption of *smf*-1 decreases biofilm-forming capacity, supporting its predominant contribution to early surface colonization. The same study identified a Cbl pilus system whose disruption also altered biofilm yield [13]. However, the available evidence does not yet establish a strictly defined stage-specific function for Cbl pili; their contribution is therefore more appropriately described as supporting subsequent biofilm development and stabilization rather than being assigned exclusively to a discrete maturation stage. Type IV pili provide dynamic surface motility and environmental sensing and are implicated in the organization and development of established biofilms. In cystic fibrosis isolates, flagellar and type IV pilus-mediated motility were not strictly required for initial biofilm formation but contributed to subsequent biofilm development [14]. Thus, *SMF-1* is most strongly associated with initial adhesion, whereas Cbl and type IV pili appear to contribute to later biofilm organization and development. Nevertheless, considerable functional overlap exists among these surface structures, and their precise stage-specific roles remain incompletely resolved in *S. maltophilia*.

### 2.2. Biofilm Maturation: Contributions of EPS, Proteins, and Extracellular DNA

Lipopolysaccharide (LPS), together with exopolysaccharides, contributes to the physicochemical properties of the *S. maltophilia* cell surface and is functionally linked to biofilm-associated phenotypes. LPS consists of lipid A, a core oligosaccharide, and an O-antigen region [15], and structural studies have characterized several distinctive sugar constituents in *S. maltophilia* [16,17]. More importantly for biofilm biology, genetic studies have linked the LPS/exopolysaccharide-associated genes *rmlA*, *rmlC*, and *xanB* to changes in surface-associated behavior [18]. Disruption of these genes alters LPS architecture and is accompanied by changes in swimming or twitching motility, surface attachment, and biofilm yield. Notably, the magnitude and direction of these biofilm phenotypes can vary with the growth surface, indicating that these genes do not simply function as uniform positive regulators of biomass accumulation. Altered LPS/EPS biosynthesis may influence biofilm development through changes in cell-surface physicochemical properties and outer-membrane organization, as well as secondary effects on surface appendages such as flagella and type IV pili. Thus, the relationship between LPS and biofilm formation is better understood as a coordinated effect of envelope architecture, motility, adhesion, and extracellular polysaccharide production rather than as an effect of any single LPS monosaccharide component [19].

Extracellular DNA (eDNA) is an important structural component of the extracellular matrix in many bacterial biofilms. However, direct experimental evidence defining its structural or regulatory requirement in monospecies *S. maltophilia* biofilms remains limited. Evidence involving *S. maltophilia* has largely been obtained from mixed-species systems. For example, in patterned lubricant-infused surfaces containing *P. aeruginosa* and *S. maltophilia*, DNase treatment reduced the formation of inter-biofilm bridges, supporting a role for extracellular nucleic acids in maintaining the architecture of mixed microbial communities [20]. Nevertheless, these findings should not be interpreted as demonstrating an equivalent structural requirement for eDNA in monospecies *S. maltophilia* biofilms. Similarly, Z-form extracellular DNA stabilized by DNABII proteins has been identified as a structural component of bacterial biofilm matrices [21]. But this mechanism has not yet been specifically established in *S. maltophilia*. Therefore, species-specific monospecies studies are required to determine the contribution of eDNA to *S. maltophilia* biofilm maturation, mechanical stability, and antimicrobial tolerance.

## 3. Regulation of Biofilm Formation by Endogenous Signaling Pathways

### 3.1. The DSF/rpf Quorum-Sensing System: A Central Axis of Biofilm Regulation

In *Xanthomonas campestris*, diffusible signal factor (DSF)-mediated intercellular signaling regulates virulence-factor synthesis and plant pathogenicity [22]. *S. maltophilia* shares close evolutionary relationships with plant pathogens from the Xanthomonas and Xylella genera, and it also carries the same DSF signal pathway. *S. maltophilia* and *Pseudomonas aeruginosa* live together in various environments, and they are often simultaneously isolated from cystic fibrosis infectious lesions. In mixed-species biofilms, *S. maltophilia* markedly alters *P. aeruginosa* biofilm morphology, inducing elongated filamentous structures. This effect depends on DSF synthesized by *S. maltophilia*. DSF and BDSF bind to the sensor kinase PA1396 of *P. aeruginosa*, change its gene expression profiles, regulate biofilm growth and raise its resistance to polymyxins. An *rpfF* mutant unable to synthesize DSF fails to induce filamentous biofilm morphology; complementation with cloned rpfF or supplementation with DSF restores this phenotype [23]. The *rpf* cluster mediates DSF synthesis and sensing in *S. maltophilia*, and clinical isolates can be divided into two major configurations, RpfC–RpfF-1 and RpfC–RpfF-2 [24]. These subtypes differ in their basal capacity to produce DSF. In RpfC–RpfF-1 strains, *rpfF*-1 is functionally active and produces detectable DSF. Consequently, deletion of *rpfF*-1 removes an active signaling input and results in measurable changes in biofilm formation, swarming motility, and virulence. In contrast, RpfC-2 constitutively suppresses *rpfF*-2 activity under the conditions examined, resulting in little or no detectable basal DSF production. Deletion of an already repressed *rpfF*-2 therefore produces a substantially weaker phenotypic effect. Accordingly, the contrasting consequences of *rpfF* deletion primarily reflect subtype-dependent differences in RpfC-mediated control and basal DSF production rather than fundamentally different biological activities of the DSF molecule itself (Figure 2a).

### 3.2. Sensing Exogenous AHL Signals: SmoR-Mediated Interspecies Communication

Standard AHL quorum-sensing systems contain LuxI synthetase and LuxR regulatory protein, and their coding genes are generally arranged adjacently on the genome. In *S. maltophilia*, preliminary genome analyses identified multiple putative luxR genes but no LuxI synthase. Smlt1839 (named SmoR), an independent LuxR protein, carries a conserved AHL binding region and helix-turn-helix DNA binding structural motif. These data reveal *S. maltophilia* utilizes the standalone SmoR protein to capture AHL signals released by surrounding bacteria, then regulates group behaviors (swarming, colonization, biofilm and virulence). By responding to AHL signals produced by other bacteria, *S. maltophilia* can participate in interspecies communication within polymicrobial biofilms, particularly when co-colonizing airways or medical device surfaces with pathogens such as *P. aeruginosa* [25]. Thus, the most directly supported function of SmoR in *S. maltophilia* is interspecies signal sensing rather than endogenous AHL quorum sensing. Broader roles attributed to AHL signaling in biofilm initiation, maturation, and community organization have been established in diverse bacterial systems, but the extent to which each of these processes is directly controlled by SmoR in *S. maltophilia* remains incompletely defined (Figure 2b).

### 3.3. The Second Messenger c-di-GMP: A Molecular Switch Between Motility and Adhesion

Cyclic di-GMP is a broadly conserved bacterial second messenger that coordinates transitions between motile and surface-associated lifestyles. In the general bacterial framework, GGDEF-domain proteins function predominantly as diguanylate cyclases that synthesize c-di-GMP, whereas EAL- and HD-GYP-domain proteins function as phosphodiesterases that degrade it [26]. Elevated intracellular c-di-GMP commonly favors surface attachment, extracellular matrix production, and biofilm-associated growth, whereas reduced c-di-GMP promotes motility and dispersal. These principles provide a useful framework for interpreting biofilm regulation in *S. maltophilia*; however, they should not be taken to imply that the complete repertoire of c-di-GMP metabolic enzymes and effectors has been experimentally characterized in this species.

In *S. maltophilia*, direct species-specific evidence remains more limited. A recent study identified c-di-GMP-related two-component regulatory systems associated with temperature-responsive control of biofilm- and protease-related phenotypes and showed that *clp* transcription is regulated primarily by LotS/LotR, with contributions from RpfC/RpfG and Sm0738/Sm0737 [27]. These findings support a link between c-di-GMP-associated regulatory circuitry and environmental adaptation in *S. maltophilia*, but the identities and biochemical functions of individual diguanylate cyclases, phosphodiesterases, receptors, and downstream c-di-GMP effectors remain incompletely resolved. Mechanisms involving Wsp pathway components [28] and YajQ-family c-di-GMP receptors [29] have been characterized in other bacterial systems and therefore provide mechanistic hypotheses rather than direct evidence for identical signaling interactions in *S. maltophilia*. Further species-specific genetic and biochemical studies are required to define the c-di-GMP signaling architecture of this organism (Figure 2c).

### 3.4. Two-Component Regulatory Systems: Transmission of Environmental Signals to Transcriptional Responses

Two-component systems are stimulus-response functional units enabling bacteria to detect environmental shifts and launch adaptive transcriptional reactions. Typically, a specific stimulus activates autophosphorylation of a sensor histidine kinase, followed by transfer of the phosphate group to a cognate response regulator, thereby initiating an adaptive response [30]. In Xanthomonas and related bacteria, the RpfC/RpfG TCS (two-component system) participates in sensing and transducing DSF quorum-sensing signals [24]. FF11 strain separated from frozen Antarctic krill relies on RpfC/RpfG to partially regulate temperature-induced SmtP protease synthesis. This network integrates DSF and temperature signals to regulate virulence-factor production, biofilm formation, and environmental adaptation. qRT-PCR (quantitative reverse-transcription PCR) analysis showed that the *rpf* system mediates approximately 60% of *clp* transcriptional activity. *Clp* is a key transcription factor linked to LotS/LotR, and together these components form a signaling network that fully controls SmtP production [31]. In *S. maltophilia*, PhoPQ contributes to multiple physiological and stress-adaptation phenotypes, including swimming motility and tolerance to oxidative, envelope, and iron-related stresses, and is required for full virulence-associated fitness [32]. However, direct evidence demonstrating that PhoPQ specifically regulates biofilm formation remains limited. Its relevance to biofilm biology is therefore currently indirect and is inferred from its effects on motility, stress adaptation, and colonization-associated physiology. Accordingly, PhoPQ should be considered a potential biofilm-modulating stress-response system rather than an established core regulator of *S. maltophilia* biofilm formation (Figure 2d).

### 3.5. Global Transcription Factors and Stress-Response Regulatory Networks

Global transcriptional regulators and stress-response pathways influence *S. maltophilia* biofilm-associated phenotypes, but the strength and mechanistic nature of the supporting evidence differ among regulators. Iron availability provides one of the clearest species-specific links. In wild-type K279a and a *fur* mutant, iron limitation or disruption of *fur* altered adhesion, extracellular polysaccharide production, biofilm accumulation, oxidative-stress responses, and virulence-associated phenotypes [33]. These findings establish a functional relationship between iron/Fur-dependent physiology and biofilm formation, although they do not imply that Fur directly transcriptionally controls all biofilm-associated genes.

Efflux-associated iron homeostasis represents an additional physiological connection. SmeYZ, SbiAB, and SmeDEF participate redundantly in stenobactin export and intracellular iron homeostasis in *S. maltophilia* [34]. These findings demonstrate a direct role in iron physiology, whereas their contribution to biofilm formation through this pathway should currently be considered indirect. Similarly, the SoxR–SodA1 pathway has direct transcriptional evidence for oxidative-stress regulation: SoxR regulates *sodA*1 expression and contributes to survival under oxidative stress [35]. Its potential contribution to biofilm persistence is therefore mechanistically plausible but should be distinguished from direct evidence of biofilm-specific transcriptional regulation.

In contrast, FsnR has direct genetic evidence linking it to *S. maltophilia* biofilm formation. Genome-wide transposon screening identified FsnR as an orphan response regulator required for normal flagellar assembly, motility, and biofilm-associated phenotypes [36]. FleQ is also present in *S. maltophilia*, and its regulatory domain has been structurally characterized; however, the c-di-GMP-dependent matrix-regulatory functions described for FleQ in other bacteria should not be assumed to be fully established in *S. maltophilia*. Overall, these regulators converge functionally on processes relevant to biofilm formation, including iron homeostasis, oxidative-stress adaptation, and motility, but they should not be interpreted as components of a fully resolved linear regulatory cascade (Figure 2e and Table 1).

## 4. Coupling Between Efflux Pumps, Antimicrobial Resistance, and Biofilm Formation

### 4.1. Efflux Pumps and Biofilm-Associated Phenotypes

Various drug resistance mechanisms are the primary reason for unsuccessful infection therapy. Multidrug efflux pumps are membrane proteins that discharge antibiotics, divided into five main types: RND, MFS (major facilitator superfamily), SMR (small multidrug resistance), ABC and MATE (multidrug and toxic compound extrusion) transport families [28].

Apart from discharging antibacterial drugs, RND efflux transporters are now proven to link bacterial physiological activities and virulence traits [37]. Genome annotation of the K279a strain predicted eight RND efflux systems: SmeABC, SmeDEF, SmeGH, SmeIJK, SmeMN, SmeOP, SmeVWX and SmeYZ [38]. The strength of evidence linking individual efflux systems to biofilm formation varies substantially. SmeYZ and MacABCsm have species-specific experimental evidence demonstrating biofilm-associated phenotypes in addition to their antimicrobial-efflux functions [39]. Wu et al. further described a notable function of SmeYZ, SbiAB, and SmeDEF in the redundant secretion of siderophores [34]. Celastrol-mediated suppression of *smeYZ* expression is also associated with inhibition of biofilm formation, providing pharmacological support for a relationship between SmeYZ-associated physiology and the biofilm phenotype [40]. By contrast, SmeDEF and SmeIJK are more directly supported as contributors to iron homeostasis, membrane integrity, or envelope-stress responses [41]. Their relevance to biofilm development is therefore more appropriately considered indirect unless a specific biofilm phenotype has been experimentally demonstrated. Accordingly, physiological association with biofilm-supporting processes should not be interpreted as evidence that every efflux pump directly regulates biofilm formation (Table 2).

### 4.2. Mechanisms Underlying Enhanced Antibiotic Tolerance in Biofilm-Associated Cells

Antimicrobial resistance, biofilm-associated tolerance, and persistence are related but mechanistically distinct phenomena. Resistance refers to a heritable capacity to grow at antimicrobial concentrations that inhibit susceptible bacterial populations and is commonly reflected by an increased MIC. In contrast, tolerance refers to the ability of a bacterial population to survive transient antimicrobial exposure without necessarily exhibiting an increased MIC, whereas persistence describes survival of a small phenotypically tolerant subpopulation during otherwise lethal antimicrobial treatment. Accordingly, the reduced antimicrobial efficacy observed in biofilms frequently reflects tolerance and persistence in addition to genetically encoded resistance mechanisms.

A comparative experiment tested seven fluoroquinolones, cotrimoxazole and ceftazidime for their anti-biofilm capacity. The strains rapidly adhered to surfaces and formed mature biofilms within 24 h. Biofilm architecture significantly reduced antibiotic efficacy. At subinhibitory concentrations (1/2 and 1/4 MIC), only fluoroquinolones slightly reduced biofilm biomass, whereas ceftazidime and SXT (trimethoprim–sulfamethoxazole) had no evident inhibitory activity against biofilms, indicating high tolerance to commonly used clinical antibiotics. Although most tested drugs reduced the viability of biofilm-embedded cells at sub-MIC (minimum inhibitory concentration) levels, none completely eradicated the biofilm population. Against mature preformed biofilms, high concentrations of bactericidal drugs produced only limited clearance: fluoroquinolones partially reduced biomass, ceftazidime completely lost biofilm-eradicating activity, and SXT was only weakly effective at extremely high concentrations [42]. In the airways of patients with cystic fibrosis, *S. maltophilia* is an emerging clinically important pathogen. Using CF (cystic fibrosis) bronchial epithelial IB3-1 cells, one study showed that the bacterium forms biofilms on epithelial surfaces and invades intracellularly, thereby evading host immune clearance and markedly increasing biofilm-associated antimicrobial tolerance. These properties contribute to persistent colonization and refractory infection in CF lungs [10]. Another study examining the impact of biofilms on antimicrobial resistance collected 51 clinical isolates and established in vitro biofilm models to compare planktonic and biofilm susceptibility. Most isolates showed multidrug resistance and biofilm-forming ability. Biofilms significantly reduced susceptibility to most antibiotics, with levofloxacin showing the strongest inhibitory effect. Monotherapy was limited, whereas erythromycin increased sensitivity to levofloxacin and cefoperazone/sulbactam; combination analysis confirmed synergistic biofilm clearance by erythromycin plus levofloxacin [43]. Biofilms not only restrict antibiotic penetration but also facilitate antibiotic inactivation [44], playing a central role in the pathogenesis of infections across multiple systems [45,46,47,48]. Therefore, targeting biofilm-associated antimicrobial tolerance and persistence represents a critical breakthrough in antimicrobial resistance research. Recent studies have demonstrated that, in addition to emerging approaches such as CRISPR/Cas9-based gene editing and nanotechnology-assisted strategies [49], the utilization of bacterial or viral components to inhibit biofilm formation represents an intriguing and effective approach [50]. However, effective clinical strategies for managing biofilm-associated antimicrobial tolerance and persistent infection in *S. maltophilia* infections remain limited [51].

Recent preclinical work has evaluated the lytic bacteriophage vB_SmaS_QH16 against *S. maltophilia* using in vitro, biofilm, and animal models [52]. Its reported antimicrobial activity, stability, and preliminary safety profile support further investigation as a potential therapeutic candidate. However, these findings remain preclinical and do not establish clinical efficacy or translational readiness. Important barriers include the emergence of phage-resistant bacterial populations, host immune neutralization, pharmacokinetic and tissue-distribution constraints, formulation and delivery challenges, comprehensive safety evaluation, and the need for controlled clinical studies. Accordingly, the current evidence should be regarded as preclinical proof-of-concept rather than demonstration of established clinical utility.

## 5. Host Interactions and Regulatory Features in Polymicrobial Biofilms

### 5.1. Host–Cell Adhesion and Biofilm Formation During Chronic Infection

*S. maltophilia* can adhere to respiratory epithelial cells, mucosal mucins, and medical device surfaces. Its biofilm-forming capacity is closely associated with chronic pulmonary infection and catheter- or implant-associated infection. Pompilio’s team tested 40 clinical strains and found most attach to polystyrene and form biofilms; surface hydrophobicity acts as a major driving factor [53]. *S. maltophilia* carries natural multidrug resistance, and its isolation rate from CF patients’ lungs has risen over the last ten years. Long-term colonization can lead to pulmonary infection [51]. The bacterium regulates biofilm formation through genes such as *smf-1*, *rmlA* (glucose-1-phosphate thymidylyltransferase), and rpfF (DSF synthase) [54]. *SMF-1* fimbriae are primarily associated with initial adhesion, whereas Cbl pili contribute to subsequent biofilm development, although their precise stage-specific function remains incompletely defined [13]. Other biofilm-related genes contain *spgM*, *rmlC* and *xanB*, which encode bifunctional phosphoglucose mutase, rhamnose epimerase and phosphomannose isomerase separately. In addition, genes controlling LPS, fimbriae and flagella synthesis, plus intracellular c-di-GMP concentration, jointly regulate biofilm generation [55]. Through adhesins, fimbriae, and flagella, *S. maltophilia* completes early colonization, and through extracellular polysaccharide synthesis and quorum-sensing regulation, it develops mature biofilms. These biofilms enable immune evasion, increase population-level antimicrobial tolerance, and contribute to chronic recurrent infection.

### 5.2. Competition, Cooperation, and Signal Interference in Polymicrobial Biofilms

Major pathogens including *P. aeruginosa*, *Staphylococcus aureus* and *Burkholderia cepacia* frequently coexist with *S. maltophilia* inside hosts collected from clinical specimens and form infectious colonies with great clinical significance [56,57]. Within polymicrobial biofilms, bacterial strains interact in diverse ways. Within polymicrobial biofilms, bacterial species may detect exogenous quorum-sensing molecules, share protective niches within the EPS matrix, and collectively benefit from a community-level resistance microenvironment. DSF produced by *S. maltophilia* induces marked morphological changes in *P. aeruginosa* biofilms within mixed-species aggregates. In dual-species biofilms, the *P. aeruginosa* two-component sensor BptS detects DSF and reduces susceptibility to polymyxin B and colistin [57]. In a mouse respiratory infection model, co-infection with *P. aeruginosa* significantly increased *S. maltophilia* burdens in bronchoalveolar lavage fluid and lung homogenates. The two species formed integrated biofilms and co-localized in infected lung tissue [58]. Conversely, the *P. aeruginosa* quorum-sensing molecule PQS (Pseudomonas quinolone signal) induces pilus-dependent aggregation in *S. maltophilia*, thereby enhancing its competitive survival within mixed communities [59]. Polymicrobial interactions are not exclusively cooperative. Bacterial species may also compete for nutrients or secrete antimicrobial factors that restrict neighboring populations. Members of the *S. maltophilia* complex can eliminate competing bacteria through the type VI secretion system, thereby promoting interspecies competition and niche exclusion [60]. Studies using dual- and triple-species biofilm systems further show that *S. maltophilia* alters the metabolic and physiological states of neighboring bacteria. Depending on species and strain composition, it frequently occupies the basal biofilm layer and contributes to the formation of stratified community structures. It also modifies gene expression in *S. aureus* and *P. aeruginosa*, shifts its own metabolism toward fermentation and anerobic respiration, and suppresses quorum-sensing-dependent virulence-factor expression in *P. aeruginosa* [61]. Thus, *S. maltophilia* can promote mutualistic coexistence through interspecies signaling and structural cooperation while also mediating antagonistic interactions through nutrient competition and contact-dependent bacterial killing. Together, these cooperative and competitive processes shape polymicrobial community organization, infection progression, and antimicrobial-resistant phenotypes.

## 6. Anti-Biofilm Intervention Strategies and Potential Targets

### 6.1. Targeting the DSF/rpf Quorum-Sensing System

Two *rpfF* subtypes, *rpfF*-1 and *rpfF*-2, have been identified in *S. maltophilia*. Only strains carrying the RpfC–RpfF-1 system produce detectable levels of DSF, which regulates biofilm formation, motility, and other virulence-associated features [24]. Both the Wsp chemotaxis pathway and the DSF quorum-sensing system modulate biofilm development through c-di-GMP signaling, with substantial cross-talk between the two pathways. Mechanistically, WspR, a diguanylate cyclase responsible for c-di-GMP synthesis, can directly interact with RpfG, a phosphodiesterase that promotes c-di-GMP degradation, thereby forming a regulatory complex. RpfG functions as an adaptor that inhibits WspR activity, reduces c-di-GMP production, and thereby suppresses biofilm development. Phosphorylation of WspR weakens this interaction and relieves the inhibitory effect. This mechanism clarifies how two signaling systems jointly regulate biofilm formation and provides potential targets for inhibiting biofilm development by disrupting pathway interactions [62]. Many pathogens use DSF-family signals to sense environmental conditions and cell density, thereby regulating virulence, biofilm development, and antibiotic tolerance. These cis-unsaturated fatty acid signals were first discovered in Xanthomonas and were later identified in pathogens such as *Burkholderia cepacia* and *P. aeruginosa.* Current studies have defined DSF biosynthetic pathways, novel synthases, receptor proteins, and intracellular signaling cascades that regulate biofilm formation. DSF also mediates interspecies communication and alters community-level behavior. Therefore, inhibiting DSF synthesis, blocking signal recognition, or disrupting signal transduction may suppress biofilm formation and reduce virulence and resistance [63]. In the rice–Xanthomonas oryzae interaction system, endogenous plant hormones bidirectionally affect host immunity and bacterial virulence. Salicylic acid activates bacterial quorum sensing, accelerates DSF production and raises polysaccharide and xanthomonadin virulence factors to aggravate infection severity. DSF signals can also regulate plant hormone-response genes. Quorum quenching has emerged as an anti-resistance strategy that blocks quorum-sensing pathways and inhibits biofilm and virulence-factor production. Major quorum-quenching approaches include quorum-sensing signal analogs and signal-degrading enzymes [64].

### 6.2. Degrading EPS and eDNA: Destabilizing the Biofilm Matrix

Disruption of the extracellular biofilm matrix using DNase, proteases, polysaccharide-degrading enzymes, and surfactants is a common approach to improve antibiotic efficacy. In bacterial biofilms, extracellular polysaccharides, proteins, and extracellular DNA constitute major matrix components and represent potential enzymatic targets [65]. In *S. maltophilia*, polysaccharide-associated genes such as *rml*, *xanB*, and *spgM* have been linked to EPS/LPS biosynthesis and biofilm-associated phenotypes [18,55]. However, direct species-specific evidence for enzymatic disruption of mature *S. maltophilia* biofilms remains comparatively limited. Iron also acts as an important signal that markedly upregulates EPS expression and promotes biofilm development [33]. Matrix-degrading enzymes can target distinct biofilm components. DNase disrupts the eDNA scaffold, proteases hydrolyze structural proteins, and polysaccharide-degrading enzymes break down exopolysaccharides. Because many of these enzymes are biodegradable and exhibit relatively low toxicity, enzyme-based biofilm control may represent an environmentally sustainable strategy [65]. Further advances in enzyme engineering and synthetic biology may expand the range, stability, and specificity of enzyme-based anti-biofilm strategies.

### 6.3. Antimicrobial Materials, Surface Modification, and Combination Therapy

Because biofilm-associated bacteria may exhibit antibiotic tolerance hundreds to thousands of times greater than that of planktonic cells, antibiotic monotherapy is often insufficient. Combining antimicrobials with distinct mechanisms of action may therefore provide greater therapeutic benefit, particularly when used together with anti-biofilm compounds, nanocarriers, anti-adhesive coatings, or antimicrobial peptides. These combination strategies may synergistically disrupt the extracellular matrix, enhance drug penetration, restore antimicrobial susceptibility, and improve treatment outcomes [66,67]. In hospital infection control, anti-adhesive and anti-biofilm modification of indwelling catheters, ventilator circuits, and implantable materials may prevent bacterial attachment and biofilm formation at their source. Such surface-based strategies are therefore important for reducing device-associated infections and improving clinical outcomes.

Importantly, these anti-biofilm strategies differ substantially in both species specificity and translational maturity. Some approaches, including DSF-targeting compounds, selected antimicrobial peptides, efflux-associated interventions, and bacteriophages, have been evaluated directly against *S. maltophilia*, whereas evidence for several matrix-degrading enzymes, nanocarriers, and anti-adhesive materials is derived predominantly from broader bacterial biofilm models. Moreover, most available evidence remains at the in vitro or preclinical stage, and clinical evidence specifically demonstrating therapeutic efficacy against *S. maltophilia* biofilm-associated infection is currently lacking. Therefore, approaches extrapolated from other bacterial systems should be considered proof-of-concept rather than clinically validated interventions. More specifically, DSF-targeting compounds, efflux-associated interventions, and antimicrobial peptides are supported mainly by in vitro *S. maltophilia* studies, whereas phage-based approaches have progressed to both in vitro and animal evaluation. By contrast, evidence for matrix-degrading enzymes, nanocarriers, and anti-adhesive surfaces is derived largely from mixed-species or broader bacterial biofilm models. To date, no anti-biofilm strategy has been clinically validated specifically for *S. maltophilia* biofilm-associated infection (Table 3).

## 7. Conclusions and Perspectives

*S. maltophilia* is an important opportunistic pathogen whose clinical impact is closely associated with strong environmental adaptability, efficient colonization of host tissues and medical devices, intrinsic antimicrobial resistance, and biofilm-forming capacity. Biofilms generate protective extracellular polymeric substance barriers that limit antimicrobial penetration, facilitate evasion of host immune clearance, and promote the emergence of metabolically inactive persister cells [73]. These features contribute to persistent infection, recurrence, and therapeutic failure. Biofilm formation therefore represents a critical entry point for understanding chronic infection and multidrug resistance in this organism.

Multiple layered regulatory factors control the whole biofilm formation process of *S. maltophilia* according to existing research. Flagella, pili, adhesins, LPS, extracellular polysaccharides, proteins, and eDNA jointly contribute to initial adhesion, matrix assembly, and maintenance of mature biofilms. *SMF-1* fimbriae, Cbl pili, and matrix-associated genes such as rmlA, rmlC, xanB, and spgM play important roles in adhesion and biofilm development [18]. At the signaling level, the DSF/rpf quorum-sensing system, exogenous AHL sensing, c-di-GMP signaling, two-component systems, and global transcription factors interact to coordinate the transitions among motility, adhesion, biofilm maturation, and dispersal. Efflux pumps not only mediate antibiotic extrusion but also influence iron homeostasis, oxidative-stress responses, membrane integrity, and signal-molecule transport. These additional functions provide physiological links between genetically encoded antimicrobial resistance and biofilm-associated tolerance, while the two phenomena remain mechanistically distinct [38].

Future studies should address biofilm phenotypic heterogeneity among clinical isolates and apply multi-omics approaches to define regulatory differences among strains from distinct sources, infection sites, and rpf genotypes [8]. More clinically relevant experimental models are also needed to improve translational relevance. These may include cystic fibrosis airway models, catheter-associated biofilm systems, polymicrobial co-culture models, and models incorporating host immune or environmental stress.

Several components of the biofilm regulatory network may serve as therapeutic targets, including the DSF/*rpf* system, c-di-GMP metabolic enzymes, two-component systems, efflux pumps, and EPS- or eDNA-associated matrix components. Future strategies may combine quorum-sensing inhibitors, efflux-pump inhibitors [74], DNase, polysaccharide-degrading enzymes, antimicrobial peptides [75], nanodelivery systems [76], anti-adhesive materials [76], and conventional antibiotics. Such combinations could inhibit bacterial colonization, disrupt the biofilm matrix, improve antimicrobial penetration, and restore antimicrobial susceptibility.

Overall, *S. maltophilia* biofilm formation is a dynamic process shaped by structural components, signaling pathways, environmental stress responses, efflux systems, and polymicrobial interactions. Elucidating this regulatory network will deepen our understanding of persistent infection and resistance development while providing a theoretical basis for developing novel anti-biofilm strategies and optimizing clinical treatment.

## Figures and Tables

**Table 1:** Core signaling pathways regulating biofilm: key molecules and functions.

Signaling Pathway Type	Core Signal/Molecule	Key Genes/Proteins	Regulatory Effects on *S. maltophilia* Biofilm	Evidence Level/Basis
DSF/*rpf*	DSF signaling molecules	*rpfF*, *rpfC*, *rpfG*	Divided into *rpf*-1/*rpf*-2 subtypes; RpfF negatively regulates biofilm in *rpf*-1 strains; modulates motility, proteases, and antibiotic tolerance	Direct genetic and phenotypic evidence; in vivo support
AHL Exogenous Signal Sensing (Orphan LuxR)	Exogenous AHL (not self-produced)	SmoR (Smlt1839)	Sense AHL from surrounding bacteria; regulate swarming motility, colonization, biofilm, and virulence	Direct molecular and phenotypic evidence in *S. maltophilia*
c-di-GMP Second Messenger	c-di-GMP	GGDEF synthases, EAL/HD-GYP degrading enzymes, Clp	High concentration: promotes attachment, EPS production, and mature biofilm; low concentration: drives motility and biofilm dispersal; integrates temperature, QS, and TCS signals	Partial direct evidence; specific effectors partly inferred from homologous systems
Two-Component Regulatory System (TCS)	Histidine kinase (HK) + Response regulator	RpfC/RpfG, LotS/LotR, PhoPQ	RpfC/RpfG transduce DSF signals; LotS/LotR regulate Clp in a temperature-dependent manner; PhoPQ indirectly regulates colonization and oxidative stress tolerance	Direct for RpfC/RpfG and LotS/LotR; indirect biofilm relevance for PhoPQ
Global Stress Transcriptional Regulation	Iron homeostasis, ROS, σ factors	Fur, SoxR-SodA1, FleQ/FleN, FsnR	Fur deficiency/iron deficiency promotes biofilm; SoxR provides antioxidant protection for biofilm; FsnR positively regulates flagella and biofilm	Variable: genetic, transcriptional, phenotypic, and inferred evidence

**Table 2:** Efflux systems associated with antimicrobial resistance, biofilm formation, and physiological adaptation in *S. maltophilia*.

RND Efflux Pump	Basal Antibiotic Resistance Function	Biofilm/Physiological Regulatory Phenotypes	Associated Pathways
SmeYZ	Intrinsic resistance to aminoglycosides and co-trimoxazole	Promote biofilm formation, flagella assembly, oxidative stress tolerance, and extracellular protease secretion	Cooperate with SmeDEF and SbiAB to secrete siderophore stenobactin; maintain iron homeostasis
SmeDEF	Broad-spectrum antibiotic resistance	Participate in siderophore secretion; regulate matrix polysaccharide synthesis	Iron homeostasis pathway, Fur regulatory network
SmeGH	Acquired ceftazidime resistance	Negatively regulate biofilm formation	Membrane homeostasis, stress pathway
SmeIJK	Resistance to aminoglycosides and oleandomycin	Maintain cell outer membrane integrity; mediate σE envelope stress response	Oxidative stress, membrane damage repair
SmeVWX	Multidrug efflux	Regulated by SoxR; participate in biofilm maintenance under oxidative stress	ROS (reactive oxygen species) clearance pathway
MacABCsm (ABC transporter)	Resistance to macrolides, aminoglycosides, and polymyxins	Antioxidant and envelope protection; promote biofilm development	Oxidative stress tolerance network

**Table 3:** Anti-biofilm targeted strategies: targets, advantages, and limitations.

Intervention Strategy Category	Molecular Targets	Representative Approaches	Advantages	Limitations
Quorum-Sensing Quenching [68]	DSF/*rpf* pathway, SmoR-AHL sensing	DSF analogs, signal-degrading enzymes	Bacteriostatic; low resistance selection pressure; reduce virulence	Insufficient in vivo stability; weak bacteriostatic effect when used alone
Biofilm Matrix Degradation [20]	eDNA, EPS, matrix proteins	DNase, polysaccharide hydrolases, protease complexes	Directly disrupt mature biofilms; enhance antibiotic penetration	Enzymes are easily degraded by in vivo proteases; short half-life
Efflux Pump Inhibition [39,40]	SmeYZ, SmeDEF and other RND pumps	Triterpenoid celastrol, efflux pump inhibitors	Simultaneously reverse drug resistance and inhibit biofilm	Some inhibitors exhibit cytotoxicity
Medical Device Surface Modification [69,70]	Bacterial adhesion sites	Anti-adhesive coatings, SLIPS (slippery liquid-infused porous surfaces)	Prevent initial biofilm colonization; suitable for implantable devices	Coatings are prone to aging and failure in vivo over time
Novel Combination Therapies [71,72]	Multi-pathway synergy (matrix + QS + bactericidal)	Phage + antibiotics, antimicrobial peptides + enzyme preparations	Clear mature biofilms; synergistic bactericidal effect	Insufficient clinical translation data; challenging in vivo delivery

## Data Availability

No new data were created or analyzed in this study. Data sharing is not applicable to this article.
